# Supplementary material for: Hepatitis E Vaccination Preferences and Willingness-to-Pay Among Residents: A Discrete Choice Experiment Analysis
Source: Vaccines (Basel). 2025 Aug 27;13(9):906. doi: 10.3390/vaccines13090906 (PMC12474183; doi:10.3390/vaccines13090906)
Supplement: Supplementary file 1 [file vaccines-13-00906-s001.zip › vaccines-3742597-supplementary.pdf]

Table S1. Attributes and levels of included vaccines.

Number	Attributes	Levels	Mean
1	Protective efficacy	80–90%<br>90–100%	Expected effect of vaccination in avoiding hepatitis E infection.
2	Duration of protection	5 years<br>10 years<br>30 years	Expected duration of avoidance of hepatitis E infection achieved after vaccination.
3	Out-of-pocket cost	0–1000 CNY (Ref)<br>1000–2000 CNY<br>2000–3000 CNY	Payment acceptable to the individual for the full course of vaccination (3 doses in total).

Table S2. Example of Questionnaire Choice Set.

Attributes	Alternatives 1	Alternatives 2	Opt-out
Protective efficacy	80–90%	90–100%	
Duration of protection	10 years	30 years	
Out-of-pocket cost	2000–3000 CNY	0–1000 CNY	
Your choice	□	□	□
